# Applications of Mass Spectrometry to Structural Analysis of Marine Oligosaccharides

**DOI:** 10.3390/md12074005

**Published:** 2014-06-30

**Authors:** Yinzhi Lang, Xia Zhao, Lili Liu, Guangli Yu

**Affiliations:** 1 Key Laboratory of Marine Drugs, Ministry of Education, School of Medicine and Pharmacy, Ocean University of China, Qingdao 266003, China; E-Mails: langyinzhi@163.com (Y.L.); shelly0427@163.com (L.L.); 2 Shandong Provincial Key Laboratory of Glycoscience and Glycotechnology, Ocean University of China, Qingdao 266003, China

**Keywords:** marine oligosaccharides, ESI-MS, MALDI-MS, structural analysis

## Abstract

Marine oligosaccharides have attracted increasing attention recently in developing potential drugs and biomaterials for their particular physical and chemical properties. However, the composition and sequence analysis of marine oligosaccharides are very challenging for their structural complexity and heterogeneity. Mass spectrometry (MS) has become an important technique for carbohydrate analysis by providing more detailed structural information, including molecular mass, sugar constituent, sequence, inter-residue linkage position and substitution pattern. This paper provides an overview of the structural analysis based on MS approaches in marine oligosaccharides, which are derived from some biologically important marine polysaccharides, including agaran, carrageenan, alginate, sulfated fucan, chitosan, glycosaminoglycan (GAG) and GAG-like polysaccharides. Applications of electrospray ionization mass spectrometry (ESI-MS) are mainly presented and the general applications of matrix-assisted laser desorption/ionization mass spectrometry (MALDI-MS) are also outlined. Some technical challenges in the structural analysis of marine oligosaccharides by MS have also been pointed out.

## 1. Introduction

**Figure 1:**
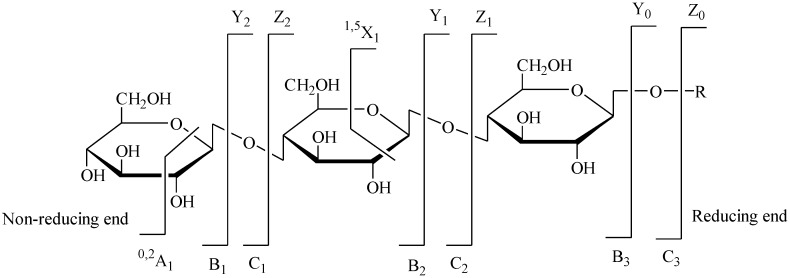
Systematic nomenclature for oligosaccharide fragmentations generated by tandem MS [15].

Marine polysaccharides and oligosaccharides have been considered recently as extraordinary resources for the discovery of functional foods [1], biochemical pharmaceuticals [2,3,4], cosmetics [5,6] and biomaterials [7,8,9]. Marine oligosaccharides are generally obtained by the hydrolysis of polysaccharides, which are mainly extracted from seaweeds and other sea creatures. Marine polysaccharides are mainly classified into three types according to their different sources: plant polysaccharides [10], animal polysaccharides [11] and microbial polysaccharides [12]. The chemical compositions and structures of most marine polysaccharides and oligosaccharides are complex and heterogeneous. As a result, more research should be focused on their structural analysis in order to utilize these carbohydrates better. Mass spectrometry (MS) based on either electrospray ionization (ESI) or matrix-assisted laser desorption/ionization (MALDI) has brought major progress in carbohydrate structural analysis, due to its high sensitivity, high precision and high speed. MALDI is considered to be a useful ionization technique for analyzing low and middle molecular weight carbohydrates [13]. Accurate masses of fragment ions obtained from ESI-MS or MALDI-MS provide reproducible and reliable information for the structural characterization of glycans. There are several ways to obtain fragment ions, and collision-induced dissociation (CID) is the most commonly used method [14]. There are two types of bond cleavage in the tandem MS of oligosaccharides, including glycosidic cleavages (B-, C-, Y-, Z-type) and cross-ring cleavages (A- and X-type), as shown in Figure 1 [15].

Some valuable reviews [16,17,18,19,20,21,22,23] emphasized the major contributions of ESI-MS or MALDI-MS to the structural elucidation of carbohydrates. However, there are few reviews focused on the MS analysis of marine oligosaccharides so far. Here, we present an overview on this topic to cover recent advances in this field with an emphasis on oligosaccharides derived from some important marine polysaccharides, including agaran, carrageenan and alginate extracted from marine algae and sulfated fucan, chitosan, glycosaminoglycan (GAG) and GAG-like polysaccharides extracted from marine animals. It is assumed that the reader is familiar with the basic principles of the ESI and MALDI process and with the instrument set up [24].

## 2. MS for Characterizing Galactan Oligosaccharides from Red Algae

Marine galactans, such as agaran and carrageenan, are abundant in red algae. The rheological aspects of the gelling or thickening properties presented by several of these polysaccharides are useful in the food industry [25,26]. Moreover, sulfated galactans have shown antiviral properties against enveloped virus [27,28]. These polymers consist of alternating (1→3)-linked β-d-galactopyranose (G) and (1→4)-linked α-d/l-galactopyranose (D) units. They are classified into agaran (l-enantiomer) and carrageenan (d-enantiomer) according to the stereochemistry of the 4-linked units [29]. They are different in the sulfation patterns and in the replacement of the D units by 3,6-anhydro-α-d/l-galactopyranose form (A), and so, their fragmentation patterns are also different in ESI tandem MS. Gonçalves found that there were diagnostic differences among the fragmentations of positional isomers of sulfated oligosaccharides derived from carrageenans and agarans [30]. Sequence determination of galactan oligosaccharides with homogeneous disaccharide compositions and heterogeneous compositions were successfully carried out by negative mode ESI-MS together with CID MS/MS [31,32,33].

### 2.1. MS of Carra-Oligosaccharides and Neocarra-Oligosaccharides

Carrageenans are normally classified into at least 15 different structures according to the presence of a 4-linked α-d-galactopyranose or 4-linked 3,6-anhydro-α-d-galactopyranose and the different sulfate contents and substitutions [34]. The main three types of carrageenans are Kappa (κ-), Iota (ι-) and Lambda (λ)-carrageenan. Neocarra- and carra-oligosaccharides, which were obtained from enzyme digestion or mild acid hydrolysis of κ-carrageenan, respectively, were used to investigate the fragmentation patterns by negative-ion ESI-MS and CID MS/MS (Table 1) [31]. It has been concluded that negative-ion detection for CID MS/MS is hindered, with sulfate being lost by expulsion as HSO_4_^−^, NaSO_3_ or NaHSO_4_, and sodium adduction to the molecule was attempted to prevent the sulfate loss for the sequence analysis of carrageenan oligosaccharides.

**Table 1:** Carrageenan oligosaccharides used for ESI-CID MS/MS [31]. (Biose units marked with a Greek letter refer to carrabiose G-A sequence derived from acid hydrolysis, whereas the primed biose units refer to the neocarrabiose A-G derived from carrageenase digestion. A, 1,4-linked-3,6-anhydro-α-d-galactopyranose; G, 1,3-linked-β-d-galactopyranose; G4S, 1,3-linked-4-sulfated-β-d-galactopyranose; A2S, 1,4-linked-2-sulfated-3,6-anhydro-α-d-Galactopyranose.)

Oligosaccharides	Assignment		Nominal Molecular Masses	
	Monosaccharide	Biose	Free Acid	Na Salt
Neocarra-tetra-1S	A-G-A-G4S	β′-κ′	710	732
Neocarra-hexa-3S	A-G4S-A-G4S-A-G4S	κ′-κ′-κ′	1176	1242
Neocarra-hexa-4S	A-G4S-A2S-G4S-A-G4S	κ′-ι′-κ′	1256	1344
Carra-tri-3S	G4S-A2S-G4S	ι-G4S	726	792
Carra-hepta-4S	G4S-A-G4S-A-G4S-A-G4S	κ-κ-κ-G4S	1418	1506

**Figure 2:**
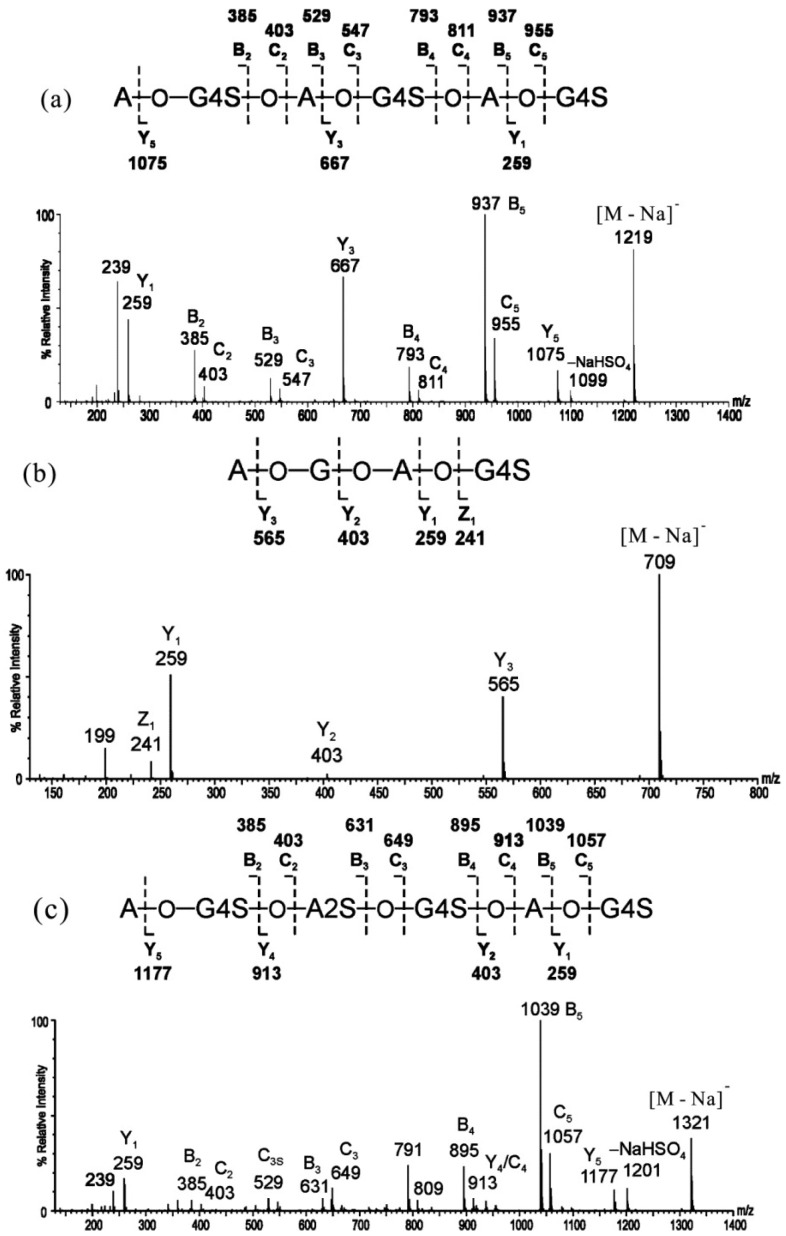
Negative-ion ESI-CID MS/MS product ion spectra of neocarra-oligosaccharides. (**a**) neocarra-hexa-3S; (**b**) neocarra-tetra-1S; (**c**) neocarra-hexa-4S. Modified from [31].

The spectra of the sodiated neo-κ-carra-oligosaccharides were characterized by extensive B- and C-ions, together with some prominent Y-ions. B- and C-type cleavages occurred at every glycosidic bond, except the non-reducing terminal residue, A, for it does not contain a sulfate to carry a negative charge. The intensity of B-ions was higher than C-ions, and Y-ions were prominent at each of the sulfated G4S residues. Sulfate loss from the main sequence ions (in the form of −120, NaHSO_4_) was at a low level (~10% of the precursor). The product ion spectrum of neocarra-hexa-3S (κ′-κ′-κ′) is shown in Figure 2a as an example. The reducing or non-reducing terminal fragment ion was assigned by the product ion spectrum of its alditol after reduction, in which the reducing terminal ions have a 2-Da increment.

The principles established in neo-κ-carra-oligosaccharides with ideal even-numbered residues were then applied to the sequence determination of unusual neocarra-oligosaccharides with hybrid disaccharide compositions. The product ion spectrum of neocarra-tetra-1S (Figure 2b, β′-κ′) showed exclusively Y-ions and a lack of B- and C-ions, due to the reducing location of the sulfate group and a non-sulfated residue, G, at an internal location. Compared with the spectrum of neocarra-hexa-3S, the main difference of neocarra-hexa-4S (Figure 2c, κ′-ι′-κ′) was the absence of the Y_3_ ion, while the B- and C-ions were similar. In addition, the losses of sulfate from the precursor, [M − Na]^−^, and fragment ion, C_3_, were apparent, due to the presence of a labile 2-O-sulfate on the residue, A. Again, the B-, C- and Y-ions were used to determine its hybrid sequence of κ′-ι′-κ′.

**Figure 3:**
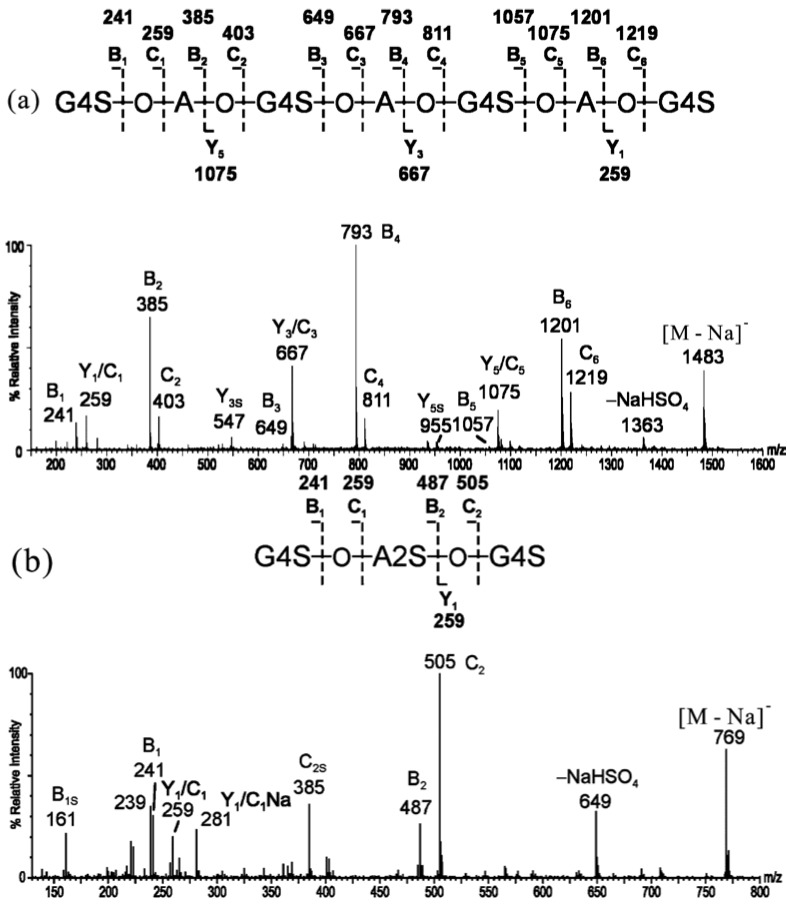
Negative-ion-ESI-CID-MS/MS product ion spectra of carra-oligosaccharides. (**a**) Carra-hepta-4S; (**b**) carra-tri-3S. Modified from [31].

The sequences of odd-numbered κ-carra-oligosaccharides were easily determined by CID MS/MS. The product ion spectrum of carra-hepta-4S (Figure 3a, κ-κ-κ-G4S) is shown as an example. Due to the symmetrical nature of the sequence, Y- and C-ions derived from the sulfated GS residues are at the same *m*/*z* values. The two types of ions were assigned by the product ion spectrum of its alditol after reduction, in which the Y ions will have a 2-Da increment. The non-reducing terminal fragment ions were dominated by the B-/C-ion doublets at non-sulfated A residues. The B-/C-ion at sulfated G4S residues turned extremely weak for the distribution of ion signals at their corresponding desulfated fragments (–NaHSO_4_, –SO_3_, –NaSO_3_ + H). Y-ions were prominent at each of the sulfated G4S residues, and the sulfate losses from the main sequence ions were observed again. Similar to the ideal odd-numbered κ-carra-oligosaccharides, the product ion spectrum of carra-tri-3S (Figure 3b, ι-G4S) mainly contained B- and C-ions. The precursor, [M − Na]^−^, and the C-ion at the residue (C_2_) tended to lose sulfates more easily, which is similar to the A2S-containing neocarra-hexa-4S.

### 2.2. MS of Agaro-Oligosaccharides

**Figure 4:**
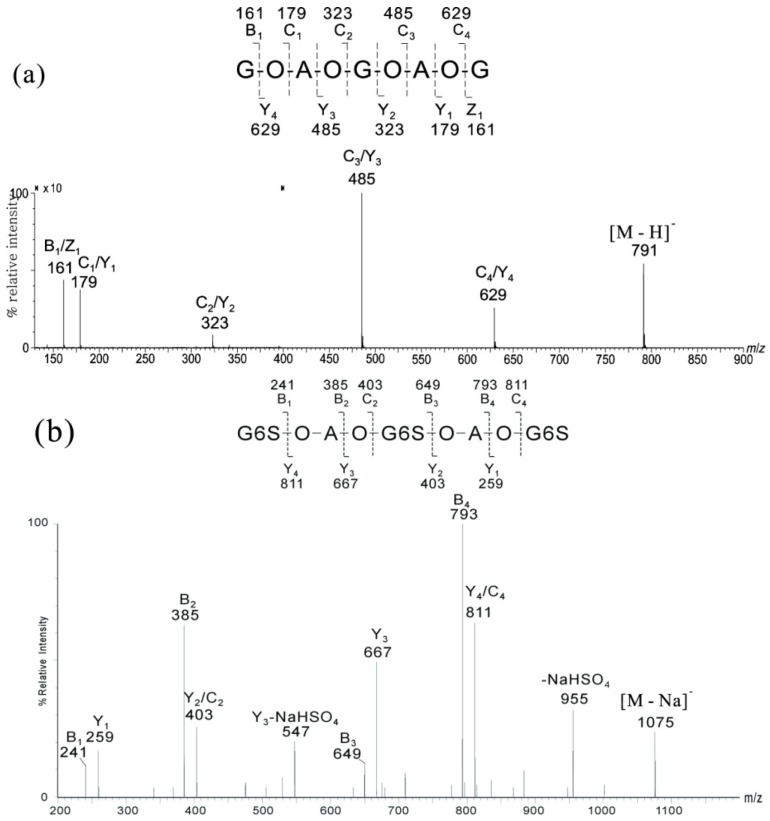
Negative-ion ESI-CID-MS/MS product ion spectra of agaro-oligosaccharides. (**a**) Agaro-pentasaccharide (modified from [32]); (**b**) 6-*O*-sulfated-agaro-pentasaccharide (modified from [33]).

Agarose (or agaran) is a neutral and linear polymer with the alternating agarobiose (3-linked β-d-galactopyranose and 4-linked 3,6-anhydro α-l-galactopyranose) sequence. Odd-numbered agaro-oligosaccharides (obtained from mild acid hydrolysis, A1–A4) were analyzed by positive-ion MALDI-MS (Table 2) to determine molecular mass, and their sequences were determined by negative-ion ESI-CID MS/MS [32]. The product ion spectrum of agaro-pentasaccharide A2 ([M − H]^−^ as the precursor) is shown in Figure 4a as an example. Clearly, the sequence was easily identified with the B_1_/Z_1_ ion at the corresponding terminal G and the C-/Y-ions at both G and A residues.

**Table 2:** Positive-ion MALDI-MS of agaro-oligosaccharides obtained by mild acid hydrolysis [32]. (A, 1,4-linked-3,6-anhydro-α-l-galactopyranose; G, 1,3-linked-β-d-galactopyranose; DP: the degree of polymerization).

Fractions	Found MNa^+^	Assignment		Theoretical MNa^+^
		DP	Sequences	
A1	509.4	3	G-A-G	509.1
A2	815.2	5	G-A-G-A-G	815.2
A3	1121.3	7	G-A-G-A-G-A-G	1121.3
A4	1427.5	9	G-A-G-A-G-A-G-A-G	1427.4
A5	1733.4	11	G-A-G-A-G-A-G-A-G-A-G	1733.5

Similar to the MS of κ-carra-oligosaccharides, the detailed sequences of highly charged odd-numbered 6-*O*-sulfated-agaro-oligosaccharides were also identified [33]. The spectrum of 6-*O*-sulfated-agaro-pentasaccharide ([M − Na]^−^ as the precursor) was characterized by extensive B and C ions, together with some prominent Y ions (Figure 4b). The non-reducing terminal fragment ions were dominated by the B-/C-ion doublets at non-sulfated A residues. The Y-type ions were prominently attributed to each fragment of G6S residues.

It is clear that the presence of the A residue is related to the unusual finding of exclusively odd-numbered oligosaccharides, which were obtained by mild acid hydrolysis of carrageenans and agarans. A two-step cleavage for the mild acid hydrolysis of A-containing galactans was proposed by Yang *et al.* through the sequence analysis of κ-carra-, agaro-, desulfated λ-carra- and ι-carra-oligosaccharides [32]. The A residue has a significant influence on the hydrolysis of galactan polysaccharides and will lead to highly specific and facile cleavage.

## 3. MS for Characterizing Alginate Oligosaccharides from Brown Algae

Alginate, an acidic linear polysaccharide existing in brown algae, consists of β-d-mannuronic acid (M) and α-l-guluronic acid (abbreviated here as G′) with exclusively 1→4 glycosidic linkages. Along its linear chain, there are homo-oligomeric regions of mannuronate (M-blocks) and guluronate (G′-blocks), as well as hetero-oligomeric regions (MG′-blocks) [35]. Alginate has several promising biomedical applications in cell immobilization, immunoisolation and as a bioscaffold, due to its ability to form temperature-independent hydrogens in the presence of calcium ions under mild conditions. The physical and chemical properties of alginate are dependent on monomer composition and distribution. The alginate sequencing therefore requires that the M and G′ epimers be distinguished, which is one of the most challenging tasks in MS analysis.

**Figure 5:**
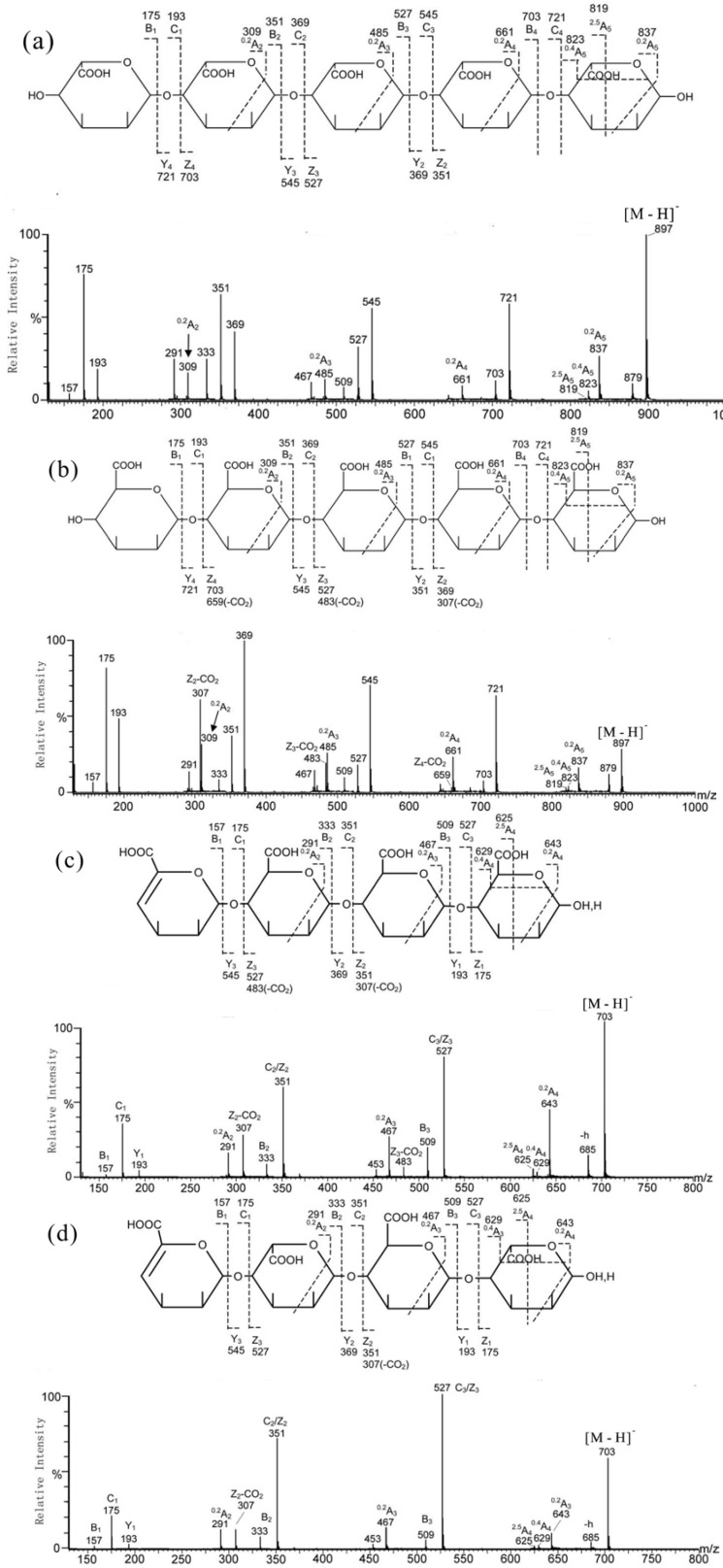
Negative-ion ES-CID-MS/MS product-ion spectra of alginate-oligosaccharides. (**a**) G′5; (**b**) M5; (**c**) ∆MMM and (**d**) ∆G′MG′. Modified from [36].

Recently, ESI-CID MS/MS has been used to determine the sequences of homo- and hetero-oligosaccharides of alginate successfully, which were obtained either by alginate lyase enzymolysis or by mild acid hydrolysis [36]. Alginate lyases cleave alginate at the hexuronic acid residue sites and release the 4,5-unsaturated hexuronic acid residue (abbreviated here as ∆) at the non-reducing terminus, while the mild acid hydrolysis produces oligosaccharide with unmodified hexuronic acid residues at both termini. The product ion spectra of homo-oligomers G′5 and M5 (Figure 5a,b) were dominated by intense B-/C- and Z-/Y-ions, together with the ^0,2^A- and ^2,5^A-ions of low intensities. Thus, their linear sequences and 4-position linkages were easily confirmed by the product ion spectra. The reducing terminal, M, was identified by the higher ratio (0.80) of [^2,5^A_5_]/[^0,4^A_5_] compared with a reducing terminal, G′ (0.15–0.2). Both non-reducing terminal M and G′ gave identical ions, B_1_ (*m*/*z* 175), C_1_ (*m*/*z* 193), together with the dehydrated and decarboxylated ions (*m*/*z* 157 and 131). They cannot be distinguished from each other at the non-reducing terminus. However, the internal M can be assigned by the presence of the unique Z_int_-CO_2_ ions (e.g., *m*/*z* 307, 483, and 659 in M5). For a longer oligosaccharide chain, the internal M residue next to the reducing terminus produced the strongest Z_int_-CO_2_ ion, whereas an internal M next to the non-reducing terminus produced the weakest.

In the analysis of hetero-oligomers with unsaturated non-reducing terminal hexuronic acid residues and mixed compositions of G′ and M, which were obtained from the lyase digestion of alginate, the same rules can also be applied. As expected, the major features of the product ion spectrum of tetrasaccharide ∆MMM (Figure 5c) are similar to those of G′5/M5, apart from the 18-Da mass shift of all the non-reducing terminal A-, B- and C-type ions, due to the unsaturated form of the terminal hexuronic acid residue. The Z_int_-CO_2_ ions and high [^2,5^A_5_]/[^0,4^A_5_] ratio (1.65) in the spectrum again were from the internal and reducing terminal M residues, respectively. In the case of ∆G′MG′ (Figure 5d), a Z_2_-CO_2_ at *m*/*z* 307 was present and Z_3_-CO_2_ was absent, indicated that M is next to the reducing terminus.

## 4. MS for Characterizing Sulfated Fucan Oligosaccharides from Echinoderm

Sulfated fucans are the most widespread sulfated polysaccharides distributed in brown algae and some marine invertebrates, particularly in sea cucumbers and sea urchins [37]. The sulfated fucans obtained from sea cucumbers have an α-(1→3)- or α-(1→4)-glycosidic linkage between 2-, 4- or 2,4-sulfated fucose residues [38,39]. They have been tested in a vast array of experimental models showing anti-coagulant, anti-tumor, immunomodulatory, anti-inflammatory and anti-complement properties [37,40]. The highly-sulfated structural characteristics of these sulfated fucans pose a challenge for sequence analysis by MS due to the lability of sulfates. Ions generated from sulfate loss of multiply sulfated oligosaccharides dominate the tandem mass spectra, and detailed structural information is rather limited [41].

**Figure 6:**
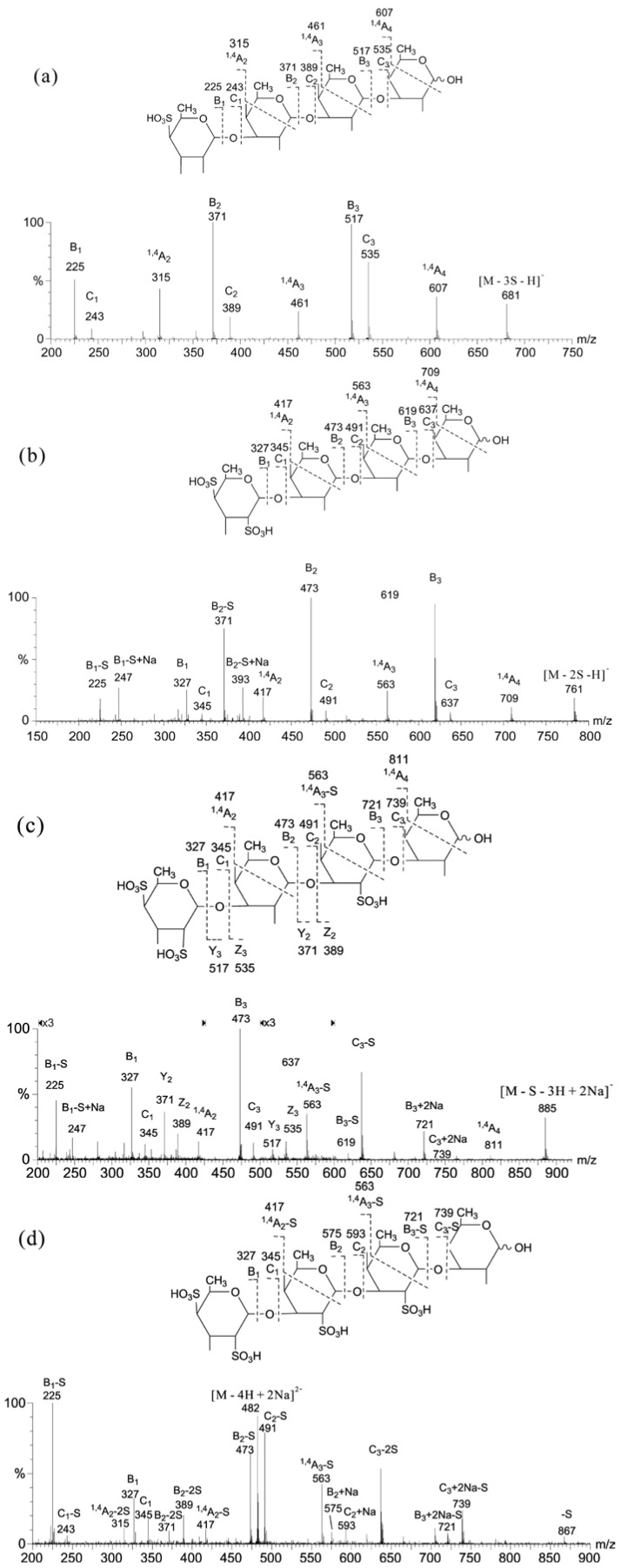
ESI-CID-MS/MS product ion spectra of tetrasaccharide using (**a**) [M − 3SO_3_ − H]^−^; (**b**) [M − 2SO_3_ − H]^−^; (**c**) [M − SO_3_ − 3H + 2Na]^−^ and (**d**) [M − 4H + 2Na]^2−^ as the precursors. Modified from [43].

It has been reported that sodiated and multiply charged molecular ions are generally more stable than the singly charged free acid form [42]. Based on this, a multi-step MS/MS strategy using different precursors to determine the sequence of the sulfated fucan oligosaccharide was established [43]. To take the tetra-sulfated tetrasaccharide obtained from sea cucumber as an example: (1) singly charged ions of the monosulfated molecule, [M − 3SO_3_ − H]^−^, were used for sequence and fucose linkage analyses; (2) [M − 2SO_3_ − H]^−^ and [M − SO_3_ − 3H + 2Na]^−^ were used for the location of the additional two sulfates; (3) the doubly charged ion of the fully sulfated and sodiated molecule, [M − 4H + 2Na]^2−^, was used for the assignment of the position of the remaining sulfate. As shown in Figure 6a, a simple product ion spectrum of [M − 3SO_3_ − H]^−^ was obtained. The full set of B/C ions were used to identify a linear sequence with a sulfate at the non-reducing terminus. Three consecutive ^1,4^A-ions, namely ^1,4^A_2_ (*m*/*z* 315), ^1,4^A_3_ (*m*/*z* 461) and ^1,4^A_4_ (*m*/*z* 607), proved that all of the glycosidic bonds are (1→3)-linked. The disulfated fragment ion, [M − 2SO_3_ − H]^−^, was then selected to assign the location of the second sulfate (Figure 6b). The B_1_/C_1_ ions at *m*/*z* 327/345 clearly identified disulfation at the non-reducing terminal fucose. The singly charged sodiated monosulfated fragment ion, [M − SO_3_ − 3H + 2Na]^−^, was further examined (Figure 6c). The mass difference of 248 Da between B_2_/C_2_ (*m*/*z* 473/491) and B_3_/C_3_ (*m*/*z* 721/739) indicated that the third sulfate is at the residue next to the reducing terminal fucose. Finally, the doubly charged ion of the fully sulfated and sodiated molecule, [M − 4H + 2Na]^2−^, was selected to assign the remaining sulfate, because doubly-charged sodiated ions are more stable, and desulfation can be largely prevented (Figure 6d). The B_2_/C_2_ (*m*/*z* 575/593) indicated that the fourth sulfate is at the residue next to the non-reducing terminal fucose. The sequence of the tetrasulfated tetrasaccharide can be assigned as: Fuc (S_2_) 1→3Fuc (S) 1→3Fuc (S) 1→3Fuc.

## 5. MS for Characterizing Chitosan Oligosaccharides from Crustaceans

Chitin is a natural polysaccharide mainly extracted from crustaceans, such as crab and shrimp shells [44]. *N*-Deacetylation of chitin under alkaline conditions [45] or by enzymatic hydrolysis generates chitosan [46]. Since *N*-deacetylation is almost never complete, chitosan is a linear polysaccharide composed of β-(1,4)-linked-d-glycosamine (abbreviated here as D′) and/or *N*-acetyl-d-glycosamine (abbreviated here as A′). Chitin and chitosan are of commercial interest, because of their high nitrogen content and their excellent properties, such as biocompatibility, biodegradability, non-toxicity and adsorptive abilities [47,48,49,50]. They can be processed into various products, including hydrogels, beads, membranes and sponges. Chitosan oligosaccharides, also named chitooligosaccharides (CHOs), composed exclusively of either D′ or A′, are named homo-CHOs, whereas those containing both monosaccharide units are named hetero-CHOs. Analysis of homo-CHOs by MS is rather straightforward, because their structures are easily deduced from their molecular mass. For isomeric hetero-CHOs, the ESI sequential MS (MS*^n^*, *n* = 2, 3 or even 4) can be applied to obtain their sequence information.

Recently, the sodiated molecules of six constitutional tetrasaccharide isomers of D′_2_A′_2_ (A′A′D′D′, A′D′D′A′, A′D′A′D′, D′A′D′A′, D′A′A′D′ and D′D′A′A′) were investigated systematically by positive-ion ESI-MS*^n^* [51]. Cross-ring ^0,2^A_4_ and ^2,4^A_4_ fragments were expected as [M − 59 + Na]^+^ and [M − 119 + Na]^+^ for a reducing end D′ and as [M − 101 + Na]^+^ and [M − 161 + Na]^+^ for a reducing end A′ residue, respectively. Cleavages of glycosidic bonds of the six isomeric tetrasaccharides gave a series of two trisaccharide fragments of the D′A′_2_ or D′_2_A′ compositions. Three disaccharide fragments of the D′_2_, D′A′ or A′_2_ and two monosaccharide fragments of the D′ or A′ were also found, respectively. The C_3_- and Y_3_-type ions are of the same D′_2_A′ composition for A′D′D′A′ and D′A′_2_ for D′A′A′D′, but they are different from the other four isomers. The C_3_-type ions of A′A′D′D′ and A′D′A′D′ have the composition of D′A′_2_, whereas the C_3_-type ions of D′D′A′A′ and D′A′D′A′ have the composition of D′_2_A′. The corresponding Y-type ions appeared as matching peaks with an increment or decrement of 42 Da, respectively.

**Figure 7:**
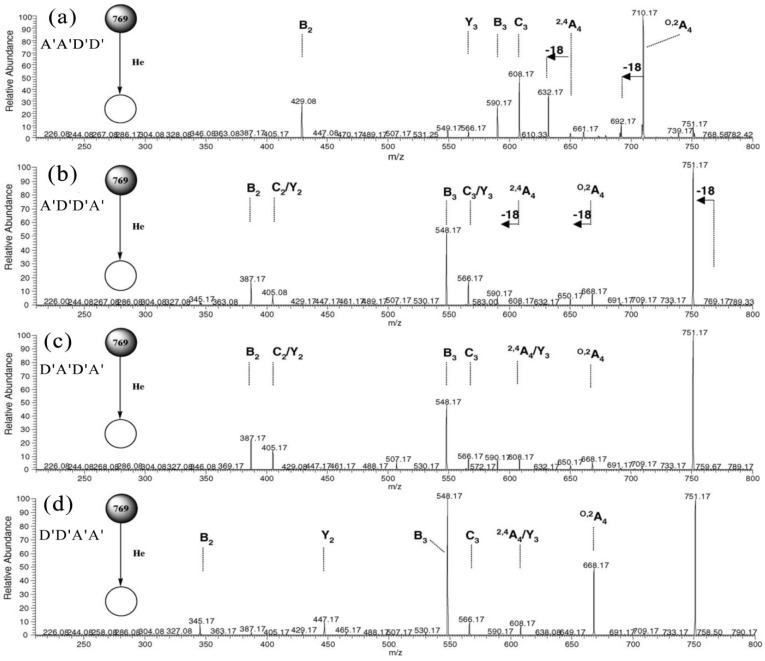
MS^2^ of the ion *m*/*z* 769 [M + Na]^+^ for (**a**) A′A′D′D′; (**b**) A′D′D′A′; (**c**) D′A′D′A′; and (**d**) D′D′A′A′. Modified from [51].

**Figure 8:**
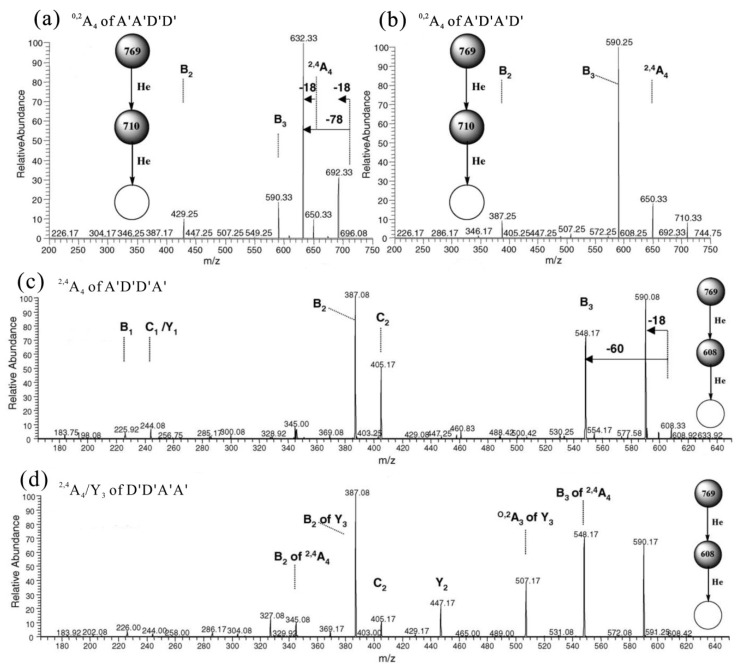
MS^3^ scan *m*/*z* 769 [M + Na]^+^ → *m*/*z* 710 [^0,2^A_4_ + Na]^+^ → products for (**a**) A′A′D′D′ and (**b**) A′D′A′D′ MS^3^ scan *m*/*z* 769 [M + Na]^+^ → *m*/*z* 608 [^2,4^A_4_ + Na]^+^ → products for (**c**) A′D′D′A′ and (**d**) D′D′A′A′. Modified from [51].

Based on the analysis of various types of cross-ring and glycosidic bond fragmentations, the following rules were established. (1) A reducing end of the D′ residue gave mainly the ^0,2^A*_n_* fragment ion [M − 59 + Na]^+^, whereas ^2,4^A*_n_* fragments [M − 119 + Na]^+^ were minor (Figure 7a); (2) A reducing end of the A′ residue preferentially gave [M − 18 + Na]^+^, and ^0,2^A*_n_* fragment ions appeared with lower abundances (Figure 7b). The assignment of ^2,4^A*_n_* is ambiguous when there was a D′ residue located at the non-reducing end (Figure 7b,c); (3) The ^0,2^A*_n_* fragment ion of a reducing end of the A′ residue was relatively more abundant when the next neighbor was A′ as compared to D′ (Figure 7c,d); (4) The ion abundances for cleavage in MS^2^ decreased generally in the order of B > C > Y, with the exception of -D′-O-D′-, where C > B > Y, (Figure 7); (5) The MS^3^ of ^0,2^A*_n_* and ^2,4^A*_n_* fragment ions yielded abundant B_n−1_ ions when the next neighbor was A′ (Figure 8b,d); however, further fragmentation by the loss of water and formaldehyde occurred when the next neighbor was D′ (Figure 8a,c).

## 6. MS for Characterizing Glycosaminoglycans Oligosaccharides from Marine Animals

Glycosaminoglycans (GAGs) are heteropolysaccharides defined by a repeating disaccharide unit without branched chains, in which one of the two monosaccharides is always an amino sugar, either *N*-acetyl-β-d-galactosamine (GalNAc) or *N*-acetyl-β-d-glucosamine (GlcNAc), and the other one is a uronic acid, either β-d-glucuronic acid (GlcA) or α-l-iduronic acid, or the other one is a galactose in place of uronic acid. There are four major classes, namely hyaluronan (HA), heparin/heparan sulfate (HP/HS), keratan sulfate (KS) and chondroitin/dermatan sulfate (CS/DS) [52]. GAGs are found in not only mammalians, but also in marine invertebrates. From a marine perspective, CS/DS is the most commonly used GAG for its abundance in shark [53] and skate (ray) resources [54]. CS/DS consists of repeating disaccharide units of HexA (β/α1→3) GalNAc (β1→4). Many types of CS exist; however, the three main classifications of CS found in higher animals include CS type A (CSA), CS type B (CSB, otherwise known as DS) and CS type C (CSC), with other rare CS/DS glycoforms existing. CS/DS has been used clinically for the treatment of chronic diseases, such as degenerative arthritis, cirrhosis and chronic photo damage [55,56,57]. The lability of sulfates, the different linkage type and the presence of uronic acid make the sequence analysis of CS/DS oligosaccharides by MS extremely challenging. One challenge is related to the CID sequencing principles, which are required for the reliable determination of sulfation sites, such as sufficient cleavage of the glycosidic bond, while keeping the SO_3_ attached. Another challenge is to determine sequences, including the linkage pattern and the uronic acid epimerization.

### 6.1. ESI-MS of CS/DS-Oligosaccharides

The ESI tandem MS has been used to confirm the sequence of the CS/DS oligosaccharides and the position of sulfate groups in GalNAc residues. For example, unsulfated chondroitin dissociated to form C-ions exclusively, while CS produced major B-/Y-ions with weak C-/Z-ions [58]. Various collision energies were also used to dissociate precursor molecules having diverse charge states in ESI tandem MS, and various fragmentations of the CS/DS oligosaccharides were observed [59]. Take the fragment ions from a saturated tetrasaccharide, [GlcA(β1→3)GalNAc4S(β1→4)GlcA(β1→3)GalNAc4S], as an example. The data showed that the complexity of fragmentation increased as the collision energy decreased. The precursor fragmented minimally at −10 V collision energy. The glycosidic cleavage ions (Y_1_^1−^, Y_3_^2−^, B_3_^1−^) and the precursor were observed in moderate relative abundance at −20 V collision energy. The precursor was absent, and the Y_1_^1−^ ion was the most abundant ion at −30 V collision energy. The best collision energy for the dissociation of the precursor is important for CS/DS oligosaccharide sequencing by producing glycosidic cleavage ions. Differences of fragmentation patterns for singly, doubly and multiply charged ions were also observed for their different conformations.

**Figure 9:**
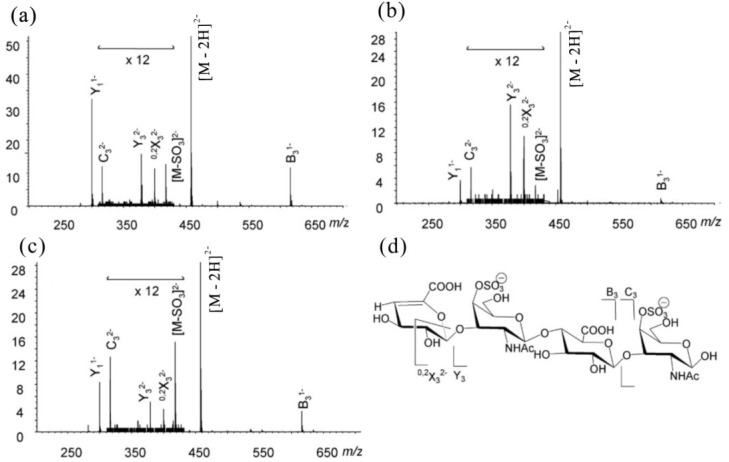
Tandem mass spectra of ∆-unsaturated dp4 derived from: (**a**) CSA; (**b**) CSB; and (**c**) CSC. The structure of ∆-unsaturated dp4 from CSA is shown in (**d**) with product ion assignments. Modified from [60].

Based on the fragmentations in ESI tandem MS, Miller found that the product ion abundances reflect the sulfation position at GalNAc residues and the epimerization of HexA residues [60]. Percent total ion abundances of signature ions of three commercial CS/DS standards, namely CSA [→4)HexA (β1→3)GalNAc4S(β1→]*_n_*, CSB [→4)IdoA(α1→3)GalNAc4S(β1→]_n_ and CSC [→4)GlcA(β1→3) GalNAc6S(β1→]_n_, are shown in Table 3. The tandem mass spectra of ∆4,5-unsaturated tetrasaccharides of CS standards are shown in Figure 9. The product ions resulting from glycosidic bond cleavages were abundant in the product ion spectra of doubly-charged precursor ions, [M − 2H]^2−^, whereas those from the losses of sulfate were in low abundance. Although the three CS/DS ∆dp4 isomers dissociated to form ions with identical *m*/*z* values, they were distinguished from each other by the differences in the abundances of six signature ions (Table 3). The ∆dp4 oligosaccharides derived from CSA were characterized by high abundances of Y_1_^1−^ and B_3_^1−^ ions (Figure 9a). CSB ∆dp4 oligosaccharides were characterized by high abundances of Y_3_^2−^ and ^0,2^X_3_^2−^ ions (Figure 9b). CSC ∆dp4 oligosaccharides were characterized by abundant C_3_^2−^ and [M − SO_3_]^2−^ ions (Figure 9c). From the differences in abundances of observed ions, we can conclude that the sulfation and epimerization positions have significant influences on the lability of certain bonds in the oligosaccharide ions.

**Table 3:** Percent total ion abundances of signature ions for ∆dp4 oligosaccharides of chondroitin sulfate (CS) type A (CSA), CS type B (CSB) and CS type C (CSC) [60].

Ion	CSA	CSB	CSC
Y_1_^1−^	33.91 ± 0.61	10.14 ± 0.56	19.88 ± 0.48
B_3_^1−^	11.22 ± 0.33	2.92 ± 0.73	7.74 ± 0.52
Y_3_^2−^	1.63 ± 0.27	3.18 ± 0.15	0.68 ± 0.05
^0,2^X_3_^2−^	0.06 ± 0.05	2.41 ± 0.31	0.44 ± 0.04
C_3_^2−^	0.96 ± 0.03	0.78 ± 0.08	2.06 ± 0.18
[M − SO_3_]^2−^	0.89 ± 0.09	0.72 ± 0.64	2.23 ± 0.05

### 6.2. MALDI MS of CS/DS-Oligosaccharides

Compared to ESI-MS, MALDI-MS produces mostly singly-charged ions for the analysis of highly acidic carbohydrates, which simplifies the data interpretation. Tremendous advances have been made for MALDI detection of alkali metal salts of sulfated carbohydrates, either by liquid crystalline matrices [61,62,63] or by the alkali metal exchange method [64]. These methods are very efficient, for the sulfate losses are partially suppressed; however, the exchange peaks of Na/H or Cs/Na are unavoidable. Therefore, it is necessary to develop a simple MALDI-MS method for the characterization of highly acidic carbohydrates.

**Figure 10:**
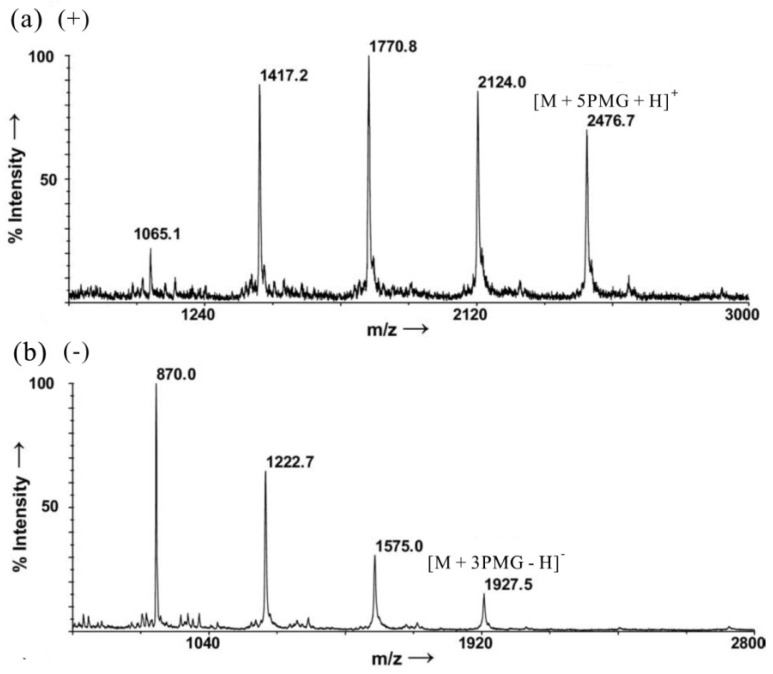
Positive-ion (**a**) and negative-ion (**b**) MALDI-MS spectra of CS–d–*tetra*–4S with pyrenemethylguanidine (PMG). Modified from [65].

Recently, a report has demonstrated how MALDI-MS can be used to obtain structural and compositional information of CS/DS simply [65]. A new powerful method for the MALDI-MS analysis of polysulfated oligosaccharides was established using pyrenemethylguanidine (PMG) as an effective derivatizing agent and ionization efficiency enhancer. Highly sulfated oligosaccharides were analyzed through the formation of complexes with PMG in both positive and negative ion modes. Unique PMG-complex ladders of peaks with various sulfation patterns were observed in the MALDI-MS spectra of CS oligosaccharides (synthetic samples, called CS-D), which are arranged in the sequence, [→4)GlcA(β1→3)GalNAc(β1→]_n_, with one sulfate group per disaccharide unit on average. An illustrative example is given in Figure 9 with a tetrasulfated CS-D tetrasaccharide. A ladder of five peaks was obtained with the fully complexed ion detected at *m*/*z* 2476.7, corresponding to the complexation of five PMG with the oligosaccharide in the positive mode (Figure 10a); while a ladder of four peaks was obtained with the highest *m*/*z* value at 1927.5 corresponding to a complexation of three PMG with the oligosaccharide in negative mode (Figure 10b). The mass differences between the observed peaks in both ionization modes correspond to a SO_3_–PMG cleavage (net loss of 353 mass units). The molecular weight of the fully-pronated analyte was therefore deduced from the spectra (Mw = 1109.7 Da). For all CS analyzed, mass spectra followed the general formula of [*n*] peaks in the negative mode and [*n* + 1] peaks in the positive mode, where *n* is the degree of sulfation of the analyte. Additionally, no sodium adducts and glycosidic cleavage fragments were detected whatsoever. This method is equally useful to analyze less sulfated CS, as well as phosphorylated CS.

## 7. MS for Characterizing GAG-Like Oligosaccharides from Marine Invertebrates

Marine GAG-like polysaccharides are a group of compounds with similar structures to the mammalian GAGs, but containing distinct sulfation patterns and/or the occurrence of branched units. Marine GAG-like polysaccharides have been exploited for their antithrombotic, anticoagulant, antimetastatic, anti-inflammatory and significant neurite outgrowth-promoting activities [66,67,68,69,70,71]. These GAG-like polysaccharides are present in several families of invertebrate animals from the phyla Cnidaria, Arthropoda, Mollusca, Echinodermata and Chordata. Generally, the GAG-like polysaccharides from marine sources contain high negative charge density due to the presence of sulfate or carboxyl groups in different positions [72,73,74,75]. In addition, unique structural modifications, such as glucose and sulfated fucose branches, which were rarely described in GAGs, are also found. These structural characteristics pose a difficult challenge for structural characterization by MS technology. Regarding the aspect of the branching pattern, Chai found that the distinctive double glycosidic D-type cleavage of a 3-linked GlcNAc and the ^0,2^A-type fragmentation from 4-linked GlcNAc can provide partial linkage information, while the series of C-type fragment ions can give the sequence information of oligosaccharides [76].

**Figure 11:**
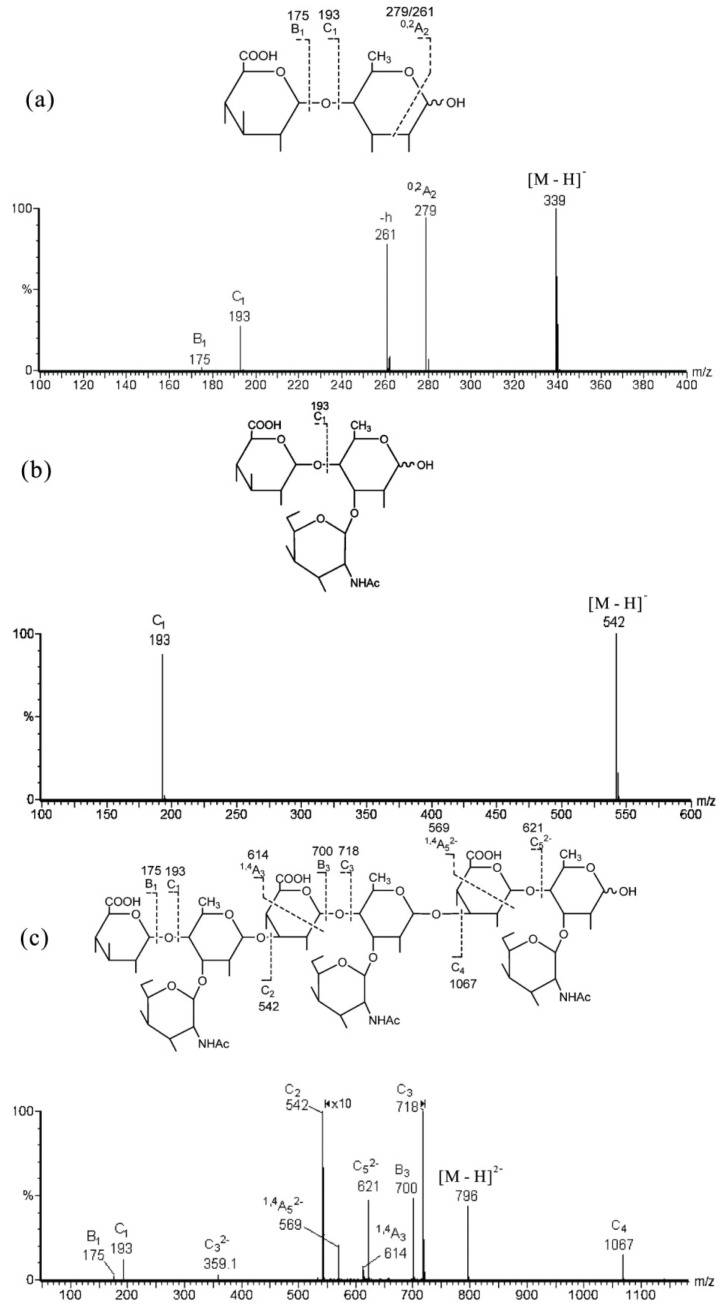
Negative-ion ESI-CID-MS/MS product-ion spectra of non-sulfated GAG-like oligosaccharides. (**a**) Disaccharide OFIVa [M − H]^−^; (**b**) trisaccharide OFIV [M − H]^−^; and (**c**) nonasaccharide OFII [M − 2H]^2−^. Modified from [77].

Recently, a novel branched non-sulfated GAG-like polysaccharide (SIP) was isolated from the melanin-free ink of squid *Ommastrephes bartrami* [77]. The sequence was determined by negative-ion ESI-CID MS/MS of its oligosaccharides (Table 4). A series of C-type ions give the sequence information, while the ^1,4^A- and ^0,2^A-type ions can provide partial linkage information, including the branching pattern. An illustrative example is given in Figure 11c with the nonasaccharide OFII [M − 2H]^−^ (*m*/*z* 796 as the precursor). The nonasaccharide gave a simple negative product ion spectrum with a full set of C-type ions, e.g., C_1_ (*m*/*z* 193, GlcA), C_2_ (*m*/*z* 542, C_1_ + Fuc + GalNAc), C_3_ (*m*/*z* 718, C_2_ + GlcA), C_4_ (*m*/*z* 1067, C_3_ + Fuc + GalNAc) and C_5_ (*m*/*z* 621.4, doubly-charged, C_4_ + GlcA), indicated a sequence of GlcA → (Fuc + GalNAc) → GlcA → (Fuc + GalNAc) → GlcA → (Fuc + GalNAc). Two weak ions, ^1,4^A_3_ at *m*/*z* 614 and doubly-charged ^1,4^A_5_ at *m*/*z* 569.4, indicated two internal 3-linkages of GlcA residues. The locations of GalNAc and Fuc were identified by comparing the product ion spectra of their disaccharide GlcA → Fuc (Figure 11a) and trisaccharide GlcA → (Fuc + GalNAc) (Figure 11b) in negative mode. The disaccharide produced a unique ^0,2^A ion, which was absent in trisaccharide. This is important to determine the 4-linked fucose in the main chain and the GalNAc substitution at the 3-position of fucose as a branch. The non-sulfated GAG-like nonasaccharide was unambiguously identified as: GlcA(β1→4)[GalNAc(β1→3)]Fuc(α1→3)GlcA(β1→4)[GalNAc(β1→3)]Fuc(α1→3)GlcA(β1→4)[GalNAc (β1→3)] Fuc.

**Table 4:** Negative-ion ESI-MS of SIP oligosaccharide fractions [77]. OFIV, the trisaccharide; OFIVa, the disaccharide; OFII, the nonasaccharide.

Fractions	Observed Ions			Assignments
	[M − H]^−^	[M − 2H]^2−^	[M − 3H]^3−^	Compositions
OFII		796.0	530.3	GlcA_3_·Fuc_3_·GalNAc_3_
OFIV	542.2			GlcA_1_·Fuc_1_·GalNAc_1_
OFIVa	339.1			GlcA_1_·Fuc_1_

## 8. Conclusions

Impressive progress has been achieved in the structural analysis of marine oligosaccharides by MS, although published data focused on the individual topic is less diverse than those described for mammalian glycans. Detailed knowledge on the fragmentation pathways of representative marine oligosaccharides, including oligosaccharides derived from agaran, carrageenan, alginate, sulfated fucan, chitosan, GAG and GAG-like polysaccharides, has been acquired by ESI-CID MS/MS and partially by MALDI-MS analysis. The analyses of the molecular mass, constituent, sequence, inter-residue linkage position and substituent distribution in marine oligosaccharide have profited from mass spectrometry.

The molecular mass and the degree of polymerization can be analyzed by ESI or MALDI-MS directly for those neutral or less sulfated oligosaccharides in free acid form. While for those highly sulfated oligosaccharides, they can be analyzed by their stabilized sodiated form in ESI-MS or deduced by derivatization in MALDI-MS. PMG derivatization used both in positive and negative ion modes can reveal the sulfation of chondroitin sulfate oligosaccharides in MALDI-MS. Various precursors of free acid, sodiated and multiply-charged molecule ions have been selected to assign the sequences and sulfate locations of highly sulfated fucan oligosaccharides; especially, an effective multi-step strategy of ESI tandem MS has been established. It is important to select the best collision energy to dissociate the particular precursor molecule in tandem MS for CS/DS oligosaccharides sequencing. Besides, characteristic the abundances of signature ions are important to determine the position of the sulfation and epimerization of CS/DS oligosaccharides.

The presence of some characteristic glycosidic and cross-ring fragmentations in ESI tandem MS at certain monosaccharide residues can provide important information on the sequence, branching pattern and specific linkage. The reducing or non-reducing terminal fragment ions can be determined by the product ion of the oligosaccharides’ alditols after reduction, in which the reducing terminal ions will have a mass increment. The ways of fragmentation of carrageenan and agaran oligosaccharides are different in their sulfation patterns and particular sequences. Epimers of mannuronate and guluronate along an alginate chain can be identified by the careful comparison of some specific fragment ions and intensity ratios, such as unique decarboxylation for an internal mannuronate residue and a higher intensity ratio of [^2,5^A]/[^0,4^A] at the reducing terminal mannuronate residue. Isomers of hetero-chitooligosaccharides can be distinguished by characteristic fragmentation ions at the reducing terminus and the residue next to the reducing terminus in positive multistage MS (MS*^n^*). The sequence of GAG-like oligosaccharides can be deduced by C-type ions and ^1,4^A ions, while the structural information of the 3-linked branch can be deduced from the additional ions observed in the MS spectra of low molecules. Differential liability of certain residues or chemical groups has also been reported for the elucidation of the depolymerization mechanisms by MS technology. With the development of MS, new techniques and approaches, further investigations with a multi-disciplinary approach are imperative for the fine structural analysis of marine carbohydrates as potential drugs and biomaterials.
